# International medical students’ experiences of psychological safety in feedback episodes: a focused ethnographic study

**DOI:** 10.1186/s12909-024-06077-8

**Published:** 2024-10-07

**Authors:** Carolyn Thomas, Shalini Gupta

**Affiliations:** School of Medicine, University of Dundee, Dundee, Scotland

**Keywords:** Medical education, International medical students, Feedback, Psychological safety

## Abstract

**Background:**

Feedback and psychological safety are well-established concepts within medical education, vital for student learning and progress. However, the concepts remain unexplored in the context of international students. This area deserves attention given the unique challenges faced by the overseas medical students due to cultural differences. The present study examines international students’ experiences of psychological safety in feedback interactions in a Scottish undergraduate medical programme.

**Methods:**

A focused ethnographic approach was adopted to explore international students’ experiences and perceptions of psychological safety in their feedback experiences. Data were collected in the form of field observations and semi-structured interviews, involving both student and faculty participants. Approximately 13hrs of fieldwork and a total of 11 interviews were conducted. These were analysed using a combination of inductive and deductive thematic analysis.

**Results:**

Data analysis identified four key themes: feedback delivery, educator attributes, cultural factors and longitudinal educational relationships. Both staff and student participants highlighted how environmental factors such as room design and group size functioned as enablers or barriers to psychological safety in feedback episodes. Additionally, students appreciated tutors who expressed vulnerability and demonstrated awareness of their cultural backgrounds. Students described significant differences between the feedback approaches in the host (UK) institute and that in their home country. Longitudinal associations fostered trust and familiarity with peers and tutors, enhancing students’ receptivity to learning and feedback.

**Conclusion:**

This present study highlights cultural differences in feedback practices across countries and their impact on psychological safety among international students. It stresses the importance of integrating overseas students by considering group dynamics, environment and diverse student needs. Staff awareness of cultural variability, openness to tutor vulnerability and fostering long-term educational relationships can greatly enhance psychological safety in learning and teaching activities. These insights are relevant amidst the growing globalisation of medical education and the mobility of students across borders, advocating for tailored integration to optimise their learning experience and achievement.

## Background

Feedback can be defined as “specific information about the comparison between a trainee’s observed performance and a standard, given with the intent to improve the trainee’s performance” [1, p.153]. As a facilitator for students’ learning, feedback is vital for students to evaluate their progress and achieve their learning outcomes. The relevance of feedback is well established in medical education and yet the feedback interactions in medical education contexts are far from perfect [2–4]. The variability in feedback across disciplines and the frequently held hierarchical and narrow perceptions by students regarding the same are only some of the issues that impact the desired effectiveness of feedback [5]. Additionally, the relationship between faculty and resident, and the style of feedback are key [6]. Existing literature suggests a preference for cultivating a partnership between students and tutors, and a departure from one-sided feedback to dialogic or interactive feedback emphasising discussion and a flexibility of roles in the conversation [4, 7]. 

Moreover, feedback practices differ across cultures and cultural factors influence students’ responsiveness to feedback. Many overseas healthcare systems, including those in Asia, follow a hierarchical structure in which doctors hold the highest authority; these doctors are also the clinical educators and the primary feedback providers engaging with students [8, 9]. These prior experiences of hierarchy and teamwork have been found to impact the working behaviours of overseas doctors when they transition to Western culture, resulting in hesitance to contest senior doctors’ opinions and voice individual concerns [10]. This is critical as students who have transferred to another higher education institution often face transitional challenges including adjustment to a new curriculum, differences in workload and balancing university responsibilities with a social life [11]. Additionally, international students have been found to attain lower grades in both written and practical assessments in comparison to their local peers [11]. 

Like most universities in the UK, the medical school at the University of Dundee hosts a multicultural cohort, with over 15% of medical students originating from countries across the world. Understanding the cultural influences in how students engage with feedback would enhance the learning experiences of international students in our school. Consultations with the local institutional experts and curriculum leads confirmed the unique struggles of these overseas students and the diversity of student needs in terms of academic support. Hence, the area of international medical students’ experiences is worth a deep analysis given the increasing globalisation in medical education and student mobility with several exchange and transfer programmes developed by medical schools across the globe [12]. 

Whilst feedback aims to evaluate and aid a learner’s development, it can often result in student demotivation and faculty stress due to fear of upsetting the other person, suggesting a lack of psychological safety (PS) in feedback conversations [3, 7, 13]. PS refers to a shared belief that the relationship is safe for interpersonal risk taking [14]. This is crucial in education as it enables students to request help, make errors without being judged and challenge senior opinions without repercussion [15]. Literature suggests that educators can foster a psychologically safe learning environment by being approachable, setting clear expectations, emphasising group learning and using non-punitive language [16–18]. In contrast, certain measures such as large group size and comparisons within student groups adversely impact PS, and in turn reduce learner motivation and participation [19–21]. However, the dynamic interactions between enablers and barriers to PS has not been investigated in international students’ and cross-cultural contexts. The present study aims to address this gap given the recent call to advance inter-cultural competence within both the migrant and host communities to ultimately enhance the experience of overseas students and healthcare professionals [22]. 

Our specific research questions were:

1. What are the experiences of international students in relation to receiving educational feedback during various teaching and learning activities in the Dundee MBChB programme?
2. What are international medical students’ understanding of psychological safety in the feedback experience?
3. What strategies could be implemented to foster and enhance PS amongst international students to maximise their learning from feedback?

## Methods

### Study Design and setting

Our ontological perspective which recognises reality as the distinct, personal view through which individuals perceive their surroundings, encouraged us to delve deeply into the human behaviours and interactions pertinent to the research objectives [23]. Employing a social constructivist paradigm, we adopted Focused Ethnography (FE) as the methodology to explore cultural aspects of our research context [24, 25]. FE was deemed suitable as it enabled the exploration of international students as cultural/ social sub-group within a short period of time using several data modalities such as observation [24, 26]. The study was conducted at the University of Dundee (UoD), focusing on international students joining the MBChB programme midway in Year 3 (of the five-year long undergraduate medical course) from two overseas partner universities in South-east Asia. Ethical approval was granted by the Institutional Ethics Committee (UOD-SMED-SLS-UG-2023-23-91) and field access was negotiated with relevant gatekeepers such as the curriculum and academic leads. Informed consent was obtained from both student and faculty participants for observations and interviews, and included consent for recording, transcribing and dissemination of information in an anonymised format.

### Sample and data Collection

Participants or key informants were chosen due to their authentic insights of cross-cultural experiences of feedback [26]. Purposive sampling of both staff and students provided a unique perspective aligning observations and interviews, acting to further enrich the data collected [26]. Overseas students were sent an email invitation via the medical school office, and those willing to participate were observed during their teaching sessions and subsequently invited for interview. A total of 25 students were eligible of which seven volunteered for both observation and interview. During fieldwork, faculty members closely involved with teaching and providing feedback for overseas students were sampled purposely and invited for interview. Around 15 staff are closely involved in teaching overseas students during their first transition year at UoD; however, not all of the dedicated teaching sessions could be observed due to time constraints. We aimed to sample a diverse selection of the specific sessions dedicated to the new transferring-in students from partner universities in Year 3 (discussed in detail below). A total of 10 staff agreed to be observed, with 4 opting to participate in interviews.

The first author (CT) conducted field observations of teaching and learning activities over a period of one month, employing a non-participant observer stance whereby the researcher is embedded in the setting but does not engage actively in the ongoing field activities [27]. Eight sessions were observed, each involving an average of 8–10 students and lasting approximately 1–2 hrs, totalling 13 hrs of observations over a one-month period. The teaching sessions that were observed included those on clinical examinations, procedural skills, Objective Structured Clinical Examination (OSCE) feedback, question and answer (Q&A) tutorials. It must be noted that these sessions were specifically designed for the group of newly transferred students and adopted a more targeted approach, bridging potential knowledge gaps and facilitating a smooth transition into the existing academic framework.

The ethnographer (CT)’s immersion in the field allowed opportunities for several informal conversations with both staff and student participants, fostering trusting relations and gleaning unfiltered perspectives in real time. We believe this further enhanced trustworthiness of our findings through generating authentic prompts, enabling capture of rich data in the interviews that followed [28]. Handwritten fieldnotes detailed the behaviours and interactions of students and staff, the setting and feedback process. These were reviewed and expanded upon within 24 h [26]. Semi-structured interviews (SSIs) lasting approximately 30–50 min were conducted with key informants on Microsoft Teams, which has the functionality of recording and generating a transcript. A brief questions outline is provided in Table 1. Data (fieldnotes, audio-records and transcripts) were managed as per the UoD data management policy [29]. 

**Table 1:** Interview guide

Initial broad questions:	• Could you tell me a bit about your recent feedback experiences at the UoD? • What aspects of these experiences have made you feel safe or supported? Why? • What aspects of these experiences have made you feel under pressure or stressed? Why? • Is there anything else that contributed to your feelings about the session?
Questions focused upon student experiences:	• How does the experience at Dundee compare to the feedback you might have received/experienced in your previous institute? • Specific clarification prompt arising during observations (different for each participant).

### Data Analysis and Team Reflexivity

Thematic analysis was conducted manually using a reflexive coding technique as per the guidance provided by Braun and Clarke [30]. Field notes and interview transcripts were evaluated through the lens of PS and reviewed using both inductive and deductive thematic analysis, to better understand the experiences and perceptions of the participants and optimise data analysis. Inductive thematic analysis allowed new insights from participants and fieldwork to be recognised as themes whilst deductive analysis utilised PS framework to derive themes. Elaborate theme discussions aided the categorisation of data into separate themes representative of and linked to the research questions [30]. Data analysis formed part of a continuous, reflexive cycle where emerging themes were identified within the data set, examined for preconceived biases or assumptions and revisited to ensure findings were faithful to the reality in the field [24, 25]. CT’s position as a medical student in the UoD proved favourable in reducing power differentials and aided in gleaning candid participant perspectives [25]. Having completed Years 1–3 of the MBChB programme at UoD, CT was undertaking a research degree comprising this project, under the supervision of the second author (SG), and therefore was separate from the cohort of medical students. We do not believe that any power imbalances arose between the researchers and participants (staff or students), owing to the negligible overlap between the two programmes, and no involvement of the researchers in any form of summative assessments for the student participants.

A reflexive diary was maintained, and research team discussions enabled questioning of assumptions around emerging findings. Moreover, method triangulation – through observations and interviews, and data source triangulation – through the diversity of research settings (described above) and participant viewpoints (staff and students) – ensured rigour [28]. Due to the time constraints of FE and inability to follow up students owing to the rotational nature of placements, member checking could not be attempted. Alternatively, subsequent observations and interviews were cross-checked through consensual validation [31]. 

## Results

Field work comprised of immersion within the field, informal discussions with informants and observations of teaching sessions, totalling to approximately 13 hrs. Subsequently, 11 interviews were conducted with key informants, involving four staff members and seven international students. A table of demographics has been included below (Table 2). An iterative process was used whereby transcripts were re-read and reviewed, generating four key themes, with interlinking subthemes related to experiences and perceptions of PS in the feedback interactions of international medical students. Detailed quotations and fieldnote extracts are included below to support the themes generated. Participants have been numbered and categorised as “S” for students or “F” for faculty to safeguard their anonymity. Certain sections of the fieldnotes and interview scripts have been masked with *** to retain confidentiality of the field sites and participants, while ensuring comprehension for the reader.

**Table 2:** Participant demographics

Participants	Male	Female
Students	4	3
Staff	3	1

### Theme 1: feedback delivery

#### Style and structure

All students described feedback received during various learning activities as constructive, with an emphasis on positive aspects of performance; this appeared to foster an open mindset amongst the feedback recipients. Echoing this, staff reported that this approach was intentional, demonstrating awareness on the potential detrimental impact of negative feedback upon students.

…if you reinforce something bad, then the student may take a good few years to get over that.

(F1)

Both staff and students expressed a preference for an interactive feedback process, stressing this as an opportunity for discussion and improved understanding.

…when you’re having a discussion when getting feedback, they [tutor] can also understand your thought process…it adds up to the overall quality of feedback.

(S1)

As feedback should focus on students’ individual performances, comparison between students was noted as contributing to an unfavourable feedback experience. One student reflected discomfort in being isolated by feedback based on their international background and the comparison with local students.

*“…we get more of the [communication skills related] feedback while the students here don’t…[I] do feel pressurised like I’m the only one who has to improve on this aspect.“*.

(S7)

#### Group size and environmental dynamics

Most participants (*n* = 7) conveyed large group size as a hindrance to the feedback process, endangering PS. Students portrayed large groups as an intimidating setting, viewing it as a barrier to feeling comfortable when receiving constructive feedback in front of others.

…it’s quite embarrassing or scary if a lot of things went wrong and all your peers are just there to listen….

(S3)

In a similar vein, faculty highlighted a large group as an obstruction to their teaching process out of concern for isolating students.

…it also increases the threat level on us as tutors because I don’t want to be seen as giving negative feedback in front of everybody….

(F2)

Room layout was documented as significant factor influencing PS and for facilitating effective feedback interactions. Both students and staff favoured small group settings for their inclusivity and comfort, particularly in mitigating power imbalances. These settings typically involved private spaces, such as rooms with closed doors, for ensuring confidentiality.

… [the table] has been set in a way where everyone’s sitting facing each other or facing the doctor and that creates a very positive learning environment.

(S6)

Whilst most sessions were conducted in small intimate groups within clinical skills classrooms, the Q&A session and OSCE feedback were held with the entire cohort of 25 newly transferred students. This necessitated a larger space, which introduced challenges in executing the session effectively. The use of a larger room, particularly one with physical obstacles, diminished the intimacy and sense of security in the teaching environment, as highlighted in fieldnote excerpt from the Q&A session below.

(Observation 2): A Q&A (Question and Answer) session.

The session takes place in the ***,* arranged in an unconventional manner: a large rectangular room with 10–12 tables*,* each equipped with 3–4 computers and chairs. At the far end of the room are the presenter desk and projector screen*,* with an additional wall-mounted screen on the side. Students are scattered throughout the room*,* some facing the tutor whilst others have their backs turned*,* focusing on the side screen instead. This appears to be rather obstructive*,* seemingly increasing the distance between the tutor and students and making it noticeably difficult for students to participate effectively in the class.*

A faculty member further stressed the importance of room design on psychological safety.

…it’s much more difficult to develop the rapport and to have that personal touch in the bigger rooms that are set out more poorly, so that in itself is going to impact on how safe the students feel.

(F4)

#### Briefing

According to participants, the session briefing was significant in fostering a respectful, safe environment. This encompassed setting class rules and normalising the idea that making mistakes is an integral part of the learning progress.

…’it’s all right to make mistakes and anything that we discuss or anything that goes wrong will stay in the room’. They [tutors] will always mention that phrase so all of us feel very safe and secure.

(S3)

Faculty also advocated for the briefing as a suitable means to enrich both teaching and feedback processes by articulating expectations and creating space for contesting viewpoints.

### Theme 2: Educator factors

#### Expressing vulnerability

Participants emphasised the value of tutors sharing their experiences, stressing that it reinforced a collaborative learning approach, diminished hierarchical barriers, and cultivated trust with students. The following fieldnote extract should convey to the reader how the tutor helped bridge the cultural gap for overseas students, alleviating fears of isolation and creating a safe space with realistic expectations of students’ academic progress.

(Observation 7): A spine and shoulder examination station.

*Concluding the session*,* the tutor shares his own personal experience with OSCEs (Objective Structured Clinical Examination) and making mistakes; the students lean forward to listen*,* brows furrowed and hands together*,* uncertain of what is about to be said. The tutor explains: ‘At a neurology station*,* I had finished what I could remember of the examination and had extra time left but knew I missed something*,* unable to pinpoint what exactly. I’m quite unsure what came over me*,* but I picked up the vibration fork and started to put it on the patient’s face! I had to be stopped by the examiner!’ The tutor laughs as he says this*,* going slightly red from re-living the moment; the students turn to one another laughing*,* unable to hide their shock. The tutor reminds them: ‘If I made that mistake and I’m still here today*,* you guys will be fine as long as you practice.’ Students appear to be relaxed and reassured by the tutor sharing this experience…*.

One student commented on finding comfort in shared experiences with their tutor, going on to say:

*”…she was also a transfer student back then so I think it just creates that feeling of they understand you.”*

(S7)

#### Body language and positioning

Participants perceived the positive impact of supportive body language, emphasising how these non-verbal cues played a crucial role in establishing an inclusive and interactive feedback environment. Tutors’ “smiling” or “clapping” appeared to reassure anxious students, boost their confidence, and encourage participation in the learning activity.

The ethnographer noted several instances (the following scene being one of many), when the tutor adopted mindful positioning and affirmative gestures to dissolve hierarchical barriers and align themselves more equally with students.

(Observation 1): A procedural skills practice session.

*I observed the tutor’s interactions with each group of students as he answered questions and explained procedures. The students*,* typically sitting in chairs at their station*,* would call over the tutor for help. I noted that he would ask the class overall*,* “Does anyone need help?” before going over to that group. When four girls asked for help with the capillary blood glucose*,* he went over and knelt beside them on the floor. At first glance*,* this may have been to ensure all students could see his demonstration clearly and to make it more inclusive. The tutor always made sure to be at an equal level with the students when giving explanations*,* rather than standing over them. The group seemed to be asking a lot of questions and engaging in discussions with the tutor.*

### Theme 3: Cultural factors

#### Transitional challenges

When asked about their cross-cultural experiences of feedback, students disclosed challenges pertaining to the transition from one institute to another, requiring significant adaptation. A fundamental difference which emerged was the focus of feedback across cultures, requiring students to adjust their educational approaches.

…we in *** [home country] mainly focus on diagnoses and the management plan. But not really how to communicate with the patient emotionally….

(S7)

Interestingly, students expressed that tutor awareness regarding cultural and institutional differences helped to reassure them, setting realistic expectations of students and enhancing feedback experiences. In contrast, as per the overseas students, the feedback approach in their home country was rather abrupt and direct which appeared to impact their PS adversely as indicated below.

.it was a very ‘we expect you to be of the highest sort of quality’ and if you don’t meet those standards, they [tutors] will tell you what is on their mind in the most direct way possible… in *** [home country], there were times where I used to go to class scared.

(S6)

#### Feedback Culture

Overall, the ethnographer consistently noted that the international students were hesitant and shy about speaking up during various learning activities. Whilst this might be misconstrued as a lack of engagement, it can also be justified as fear of getting things wrong and can be better understood considering their past experiences.

(Observation 6): A clinical skills session.

*The registrar guides the students through the examination approach*,* using case photos to illustrate key features. He intermittently poses questions to the students to seek their insights on the diagnosis and examination observations. However*,* students appear apprehensive when answering questions evidenced by a low volume and a high intonation at the end of their answers*,* perhaps demonstrating a lack of confidence and fear of getting it wrong. When students are struggling to answer*,* he uses simpler questions to extract additional information and encourage students further.*

The faculty participants also confirmed these observation findings of the overseas students being less vocal in the range of teaching and learning activities.

[international students] are perhaps happier just to listen to your feedback and tend to ask fewer questions….

(F2)

### Theme 4: longitudinal educational relationships

#### Familiarity with peers

Students frequently reported peer interactions as having a significant impact on their feedback experience, with group familiarity and consistency influencing their decision to engage and be vulnerable in sessions. Students expressed further concerns around disrupting group dynamics as new additions this academic year, emphasising the negative impact on their feedback experiences.

…if you’re not used to them [peers], you worry about how they [peers] think…then you don’t get that much out of the feedback….

(S7)

Building on this, another student provided insights into their perspectives as international students, elaborating on how they were particularly attentive to social conduct. They expressed a conscious effort to seamlessly integrate into the group, underscoring an underlying concern of making social mistakes and the potential consequence of being alienated as a result.

…so that we don’t overstep…if we’re new students then we have to take that time to bond with them and get to know them before.

(S5)

#### Familiarity with tutors

Both student and faculty participants noted how consistency and developing longitudinal relationships with tutors helped to build trust in the tutor-student relationship, serving to improve receptivity to and actioning of feedback. This established longitudinal relationship with tutors resulted in all students valuing their tutor’s dedication and attentiveness to their progress. Students regarded this as a source of motivation for learning and pivotal for fostering their professional relationship.

*“…in our first session*,* she made it a very clear point to learn each of our names… it showed that she was putting in an extra effort to sort of make me feel comfortable and that sort of helped me wanting to put an extra effort to be a better student…”*.

(S6)

Elaborating on this further, staff too conveyed that familiarity with tutors could work to alleviate pressure and enhance the safety of the learning environment.

They [students] see the same person for their dermatology sessions. So hopefully that fosters a little bit of security and reduces the stress that’s involved.

(F4)

However, it was acknowledged that consistency in tutor arrangements is not always possible; and this could lead to unfair expectations of the international students and perhaps contribute to subconscious comparisons between students.

I would imagine most tutors who are teaching just a little bit of a block won’t know who’s who and will expect the same standard of everyone….

(F1)

## Discussion

This study aimed to explore the feedback experiences of international students by examining student and faculty perceptions of psychological safety. FE enabled a detailed account of cross-cultural feedback practices to be analysed, revealing facilitators and barriers of PS in the process.

As noted in existing literature, our overseas students too were used to a pronounced hierarchical structure in their feedback encounters in their previous (home) institute [8, 9]. Moreover, through their past experiences of feedback as a unidirectional interaction, the international students in our study exemplified how historical feedback encounters mould student perceptions and limit their participation. We posit that increasing awareness regarding cultural differences in teaching and feedback context can safeguard students’ actions from misappropriation or being mislabelled as non-engagement or lacking in agency.

Our findings indicate that faculty played a vital role in mitigating transitional challenges and fostering PS by simply demonstrating an understanding of students’ previous environments and the cultural norms. Our findings resonate with suggestions by Al-Haddad and Lu [22] who emphasise the significance of host receptivity in migrants’ ability to establish bridging connections, necessitating commitment from both cultures and investment of time and resources. We found a positive shift in student behaviour over time, such as increased interaction and proactive feedback seeking behaviour, potentially indicating improved PS.

Observations in our study revealed the significance of spatial factors on the feedback and learning dynamics. The complex interaction of space in creating inclusive and non-threatening learning spaces has been discussed in literature [32, 33]. Our participants remarked that the environment and group size were crucial in setting the scene for feedback and cultivate trust between the learner and feedback provider. Based on our findings, we suggest that educators give due consideration to group sizes and the use of confidential learning spaces to enhance international students’ sense of PS in feedback encounters. However, more research is needed to understand the spatio-material dimensions in learning, and its entanglement with wellbeing and a sense of belonging [32]. 

Our student participants determined feedback to be a dialogic interaction, aiding in the understanding and application of feedback. Framing discussions collaboratively can motivate students to embrace independent learning, while explaining the rationale behind suggestions builds trust and credibility in the tutor, thus enhancing PS [34]. Additionally, participants commented on the development and consistency of longitudinal relationships between students and tutors as a facilitator for PS in feedback episodes. This aligns with Bing-You et al. [3] who emphasise the criticality of the tutor-student relationship in the progression of feedback by establishing mutual familiarity and enhanced understanding over time, enabling an effective exchange of information. Interestingly, longitudinality is established as an enabler of relationships, driving learning in several diverse medical education contexts [35]. 

Providing clarity in the outline and expectations for the session is known to alleviate learner anxiety, which can otherwise detract focus and inhibit cognitive processes [17]. However, this did not appear to be a regular practice with the tutors in our study, and we aim to reinforce the value of a detailed briefing within all learning environments to the faculty through staff development interventions. Furthermore, comparisons between students from overseas and host countries reflects an ignorance of cultural differences on the part of the tutors, and this negatively impacts PS [36]. Our findings exemplified the initial concerns of international students with adapting to foreign cultures and understanding social conduct. Overseas students eventually gained confidence in “opening up” and “settling in” within their small groups, demonstrating mutual trust and respect with peers and tutors. Time thus may be considered an enabler of PS, and the medical education community should consider building longitudinal, meaningful educational relationships to facilitate learning.

Our findings regarding the significance of tutors’ body language in fostering PS during feedback interactions resonate with some of the empirical work in different medical education contexts [16, 37]. Researchers have provided convincing explanations on how non-verbal cues can support interpersonal communication and enhance understanding [16]. Another strategy that proved effective in building PS was tutors sharing their experience of vulnerability and doubt. This approach is known to not only minimise power imbalances and build trust with students, but also offers students a realistic perspective of overcoming efforts over time, encouraging support-seeking behaviour and reducing fear of academic risks in the learning environment [17, 18]. 

### Limitations to the project

The authors acknowledge ethnographic subjectivity and endeavoured to minimise bias through reflexive journaling and triangulation-led capturing of diverse perspectives. However, absolute objectivity remains unattainable. Also, the predominance of participants from one institute were largely representative of Asian culture and restricted gathering the breadth of perspectives that potentially exist. Therefore, findings may not be generalisable to students from other geographical areas. It would be useful for future researchers to investigate feedback settings pertaining to more diverse groups of students. Additionally, PS as a concept must not be deemed as a tangible attribute that would be experienced in the same way by all individuals. Its relational and context-specific nature makes the exploration complex, and difficult to address adequately in a modest study such as this. We acknowledge the challenging terrain our work inhabits and call for larger and potentially longitudinal studies involving diverse groups of students.

## Conclusion

As described by Edmondson [15], group or team PS signifies an environment marked by common trust and respect, enabling individuals to be their authentic selves. The present study draws attention to the cultural differences in the feedback practices across different countries, and their implications for PS in various teaching and learning activities concerning international medical students. We highlight the importance of integrating overseas students into a new culture through careful considerations of group size and environment, to create safe learning spaces for feedback. Furthermore, staff awareness of the cultural variability, expressions of tutor vulnerability and cultivating longitudinal relationships with peers and faculty can significantly enhance PS amongst international students. We posit that the findings elucidated should be of interest to the medical education community given the increasing mobility of students across the borders, and the need for bespoke integration to appropriately enhance their experience and attainment.

## Data Availability

The datasets generated and/or analysed during the current study are not publicly available due to the potentially sensitive nature of the qualitative data collected and to ensure complete anonymity of student participants but are available from the corresponding author on reasonable request.

## Abbreviations

PS: Psychological Safety
FE: Focused Ethnography
MBChB: Bachelor of Medicine and Bachelor of Surgery. (Undergraduate medical degree in the UK)
SSI: Semi–structured Interviews
UoD: University of Dundee
